# Role of perioperative nursing in anesthesia: a national overview

**DOI:** 10.1590/1980-220X-REEUSP-2021-0465

**Published:** 2022-02-04

**Authors:** Cassiane de Santana Lemos, Vanessa de Brito Poveda

**Affiliations:** 1 Universidade de São Paulo, Escola de Enfermagem, Departamento de Enfermagem Médico-Cirúrgica, São Paulo, SP, Brazil.

**Keywords:** Perioperative Nursing, Anesthesia, Patient Safety, Nurse’s Role, Operating Room Nursing, Professional Practice Gaps, Enfermería Perioperatoria, Anestesia, Seguridad Del Paciente, Rol de La Enfermera, Enfermería de Quirófano, Brechas de la Práctica Profesional, Enfermagem Perioperatória, Anestesia, Segurança do Paciente, Papel do Profissional de Enfermagem, Enfermagem de Centro Cirúrgico, Lacunas da Prática Profissional

## Abstract

**Objectives:**

To assess the actions performed by the operating room nurse during anesthesia and their behavior for patient safety regarding the reporting on adverse events, and to analyze their knowledge about anesthetic practices.

**Method:**

This is a cross-sectional study carried out using an electronic questionnaire consisting of socio-demographic, professional practice, knowledge in anesthesia, patient safety, and professional practice questions, conducted from January to March 2019 with operating room nurses.

**Results:**

One hundred nurses participated, 89 (89%) being women, with a mean age of 41.09 years (SD = 9.36), time of undergraduate completion of 14.33 years (SD = 8.34). The average attendance was 4.69 operating rooms (SD = 2.07) per nurse, with an emphasis on action before induction (49; 49%). Professionals reported performance of simultaneous activities (72; 72%) and insufficient number of employees (57; 57%) as difficulties of their daily practice. Among the participants, 77 (77%) correctly cited the periods of general anesthesia and 80.4% always reported the occurrence of an adverse event.

**Conclusion:**

Nurses identified their role in anesthesia, with limitations for assistance from multiple activities and lack of professionals.

## RESUMO

**Objetivos:**

Avaliar as ações executadas pelo enfermeiro de centro cirúrgico durante a anestesia e suas condutas para segurança do paciente quanto à notificação de eventos adversos, analisar o seu conhecimento sobre as práticas anestésicas.

**Método:**

Estudo transversal realizado com questionário eletrônico composto por perguntas sócio-demográficas, prática profissional, conhecimento em anestesia, segurança do paciente e questões de exercício profissional, conduzido de janeiro a março de 2019 com enfermeiros de centro cirúrgico.

**Resultados:**

Participaram 100 enfermeiros, sendo 89 (89%) do sexo feminino, com média de idade de 41,09 anos (DP = 9,36), tempo de formação de 14,33 anos (DP = 8,34). A média de atendimento foi de 4,69 salas operatórias (DP = 2,07) por enfermeiro, com destaque para a atuação antes da indução (49; 49%). Os profissionais relataram como limitação de sua prática diária a execução de atividades simultâneas (72; 72%) e funcionários insuficientes (57; 57%). Entre os participantes, 77 (77%) citaram corretamente os períodos da anestesia geral e 80,4% sempre notificavam a ocorrência de um evento adverso.

**Conclusão:**

Os enfermeiros identificaram seu papel na anestesia, com limitações para assistência devido a múltiplas atividades e carência de profissionais.

## RESUMEN

**Objetivos:**

Evaluar las acciones realizadas por los enfermeros en el quirófano durante la anestesia y sus conductas para la seguridad del paciente en la notificación de eventos adversos, analizando sus conocimientos sobre prácticas anestésicas.

**Método:**

Estudio transversal realizado con un cuestionario electrónico compuesto por preguntas sociodemográficas, práctica profesional, conocimientos en anestesia, seguridad del paciente y cuestiones de ejercicio profesional, realizado de enero a marzo de 2019 con enfermeros de quirófano.

**Resultados:**

Participaron 100 enfermeros, 89 (89%) mujeres, con una edad media de 41,09 años (DE = 9,36), tiempo de formación de 14,33 años (DE = 8,34). La asistencia media fue de 4.69 quirófanos (DE = 2.07) por enfermero, con énfasis en la actuación antes de la inducción (49; 49%). Los profesionales informaron como limitación de su práctica diaria la ejecución de actividades simultáneas (72; 72%) y la insuficiencia de empleados (57; 57%). Entre los participantes, 77 (77%) citaron correctamente los períodos de anestesia general y el 80,4% siempre reportaron la ocurrencia de un evento adverso.

**Conclusión:**

Los enfermeros identificaron su papel en la anestesia, con limitaciones en la atención por múltiples actividades y falta de profesionales.

## INTRODUCTION

The first clinical nursing specialty in the United States of America (USA) was nurse anesthesia, especially related to the actions carried out by the anesthetist nurse Alice Magaw, who developed several works with the use of ether in anesthesia, the elaboration of the anesthetic plan, and published articles on nursing practice in anesthesia in the 19th century^(1)^.

Currently, the validation of the work of the anesthetist nurse in the United States is ensured through the Certified Registered Nurse Anesthetists (CRNA), issued by the American Association of Nurse Anesthetists (AANA), which also defines the standards of action during the anesthetic procedure, such as autonomy to define the anesthesia plan and installation of invasive devices^(2)^. The CRNA nurse shall have a degree in nursing, at least one-year experience in intensive care units, a graduate certificate in anesthesia, and approval in the national qualification exam^(3)^.

In other practice scenarios, the International Federation of Nursing Anesthetists (IFNA), an international organization created in 1989, seeks to improve anesthesia care by supporting institutions and countries that aim to develop educational standards and practices in anesthesia. IFNA recognizes the nursing work in anesthesia in several European countries, including France, Hungary, Switzerland, and Norway, together with African and Asian countries^(4)^.

With the increase in the population’s demand for health care, the reduction in the number of working professionals, and the costs related to the care provided, the development of nursing practice in anesthesia is essential for population’s access to health care. However, even in countries like the United States, which have legal recognition of the specialty, nurses face limitations in the exercise of their activities due to regional laws restricting professional practice and conflicts between medical societies for the supervision of nursing work^(4)^.

Evidence suggests lack of differences between the anesthetic procedure performed by nurses and anesthesiologists, with no difference in the quality of services provided by both professionals, which ensures the safety of the interventions proposed by the nurse during the anesthetic procedure^(5)^.

In Brazil, anesthesia is the exclusive responsibility of the anesthesiologist^(6)^. The operating room nurse works in the planning, management, execution of care and leadership of the nursing team in all pre-, trans-, and postoperative care^(7–8)^.

In the national territory there are no guidelines to govern nursing care planning during the anesthetic procedure, although a previous national study has released an instrument to direct the work of nurses during anesthesia, focusing on optimizing teamwork with the anesthesiologist^(9)^.

In this context, the justification for the study considers that the assessment of the performance of nurses in an operating room in Brazil during anesthesia can provide subsidies for understanding the facilitating and/or hindering factors for daily professional practice. Thus, this investigation aims to assess the actions performed by the operating room nurse during anesthesia and their behavior for patient safety regarding the reporting on adverse events, and to analyze their knowledge about anesthetic practices.

## METHOD

### Design of Study

This is a cross-sectional study with a quantitative approach, performed with registered nurses at the Brazilian Association of Operating Room Nurses, Anesthetic Recovery and Material and Sterilization Center (*SOBECC*).

The cross-sectional study is characterized by the assessment of the outcome and exposure of participants at the same time^(10)^.

### Population, Local, and Sample

Following project approval by the Research Ethics Committee (*CEP*), an electronic invitation via the Google forms system was sent by SOBECC to its registered email database, consisting of 4320 professionals.

From January to March 2019, nurses registered by SOBECC received a link to access an invitation letter, data collection instrument, and the Free Informed Consent Form (FICF). Professionals who agreed to participate in the study indicated their agreement by clicking “I ACCEPT”, after reading the consent form. Professionals were able to print a copy of the form, to access information about the investigation, contact of researchers, and the Research Ethics Committee (CEP).

Nurses working in the operating room, with e-mails registered in the SOBECC electronic address database, were included. Professionals who partially filled out the instrument with inconsistent information, or who reported a lack of professional experience in an operating room, were excluded.

### Data Collection

The data collection instrument consisted of 38 questions, comprising 11 sociodemographic questions, 14 questions about professional practice, six questions about knowledge in anesthesia, four questions about patient safety, and three questions about professional practice. All registered nurses received a request for completion twice and the period established for filling in and returning it was 30 days.

The data collection instrument was face-validated by three operating room specialists, with a master’s degree, who evaluated the content of the instrument regarding the criteria of clarity and relevance. The experts’ suggestions incorporated into the instrument were: description of training time and experience in the operating room, position of supervision and coordination, philanthropic work institution, work sector-outpatient operating room, technicians and nurses dedicated to anesthesia, and assembly of the room for the anesthetic procedure by the nurse resident and anesthesiologist resident.

### Data Analysis

Data were analyzed in the software R, with the description of categorical variables through absolute and relative frequency, and numerical variables through measures of central tendency (mean and standard deviation-SD).

The non-parametric Wilcoxon-Mann-Whitney test^(11)^ allows the comparison of two independent samples, through ordered categorical data, being used to compare the number of rooms per nurse and the limitations for carrying out activities due to work overload. The level of significance adopted was equal to 5%.

### Ethical Aspects

The study complied with the guidelines defined by Resolution 466/12 of the National Health Council, which regulates research involving human beings, and was approved by the Research Ethics Committee (CEP) of Escola de Enfermagem of Universidade de São Paulo (EEUSP) under opinion no. 3.081.155 on December 13, 2018.

## RESULTS

The sample consisted of 100 nurses, most of whom were female professionals (89; 89%), with a mean age of 41.09 years (SD = 9.36), 14.33 years (SD = 8.34) of education, and 11.41 years (SD = 7.49) of professional experience as well as a graduate certificate on operating rooms (62; 62%) work, and from different regions of the country, with 47 (47%) from the Southeast, 27 (27%) from the South, 19 (19%) from the Northeast, five (5%) from the Midwest, and two (2%) from the North regions.

Regarding the main function in the position, 48% of the sample consisted of clinical nurses, 26% with a coordination position, 15% supervision, 6% administrative, and 5% service management.

Regarding the workplace, 59 (59%) nurses worked in public institutions, 30 (30%) in private institutions, and 11 (11%) in philanthropic institutions. Among the sectors of activity, 77 (77%) professionals worked in the operating room and 55 (55%) worked full-time, corresponding to eight hours a day.

In professional practice assessment, 72 (72%) nurses reported that the institution in which they work did not have a care plan for nursing work in anesthesia, 28 (28%) professionals reported having a group of nursing technicians dedicated to anesthesia in their services, and in six (6%) there was an exclusive nurse to monitor anesthesia.

In the analysis of the number of operating rooms attended by nurses per shift, an average of 4.69 rooms (SD = 2.07) per professional was observed. In the analysis of care provided during periods of anesthesia, a greater participation of nurses in care was evidenced in the periods before induction (49; 49%) and during induction of anesthesia (39%) (Table 1).

**Table 1.** Nurse assistance in the operating room, according to the period of anesthesia (n = 100). São Paulo-SP, Brazil, 2019.

Nurse assistance	Period of anesthesia		
	Before induction n (%)	Induction n (%)	Reversal n (%)
In all rooms under their responsibility	49 (49)	39 (39)	23 (23)
Only for critically ill patients	31 (31)	33 (33)	32 (32)
At the call of the medical and/or nursing team	20 (20)	28 (28)	42 (42)
Never	–	–	3 (3)

As for the factors that limit or hinder assistance in the operating room during anesthesia, nurses mainly highlighted the performance of simultaneous activities (72%), insufficient staff (57%) for the surgical demand, and lack of protocols (40%) of guidance for professional practice.

Table 2 shows the nurses’ opinion regarding the professional who should check materials before induction of anesthesia. Nurses who mentioned that the conference function must be performed by nurses were responsible for a smaller number of rooms (p = 0.04) in their daily routine.

**Table 2.** Comparison between the professional category that shall check the material before induction, according to the number of rooms under the nurse’s responsibility. São Paulo-SP, Brazil, 2019.

Professional	Checking	n (%)	Number of rooms Mean (SD*)	p^†^
Nurse	Yes	38 (38)	4.18 (2.00)	0.04
	No	62 (62)	5 (2.07)	
Nursing technician	Yes	86 (86)	4.73 (2.03)	0.75
	No	14 (14)	4.43 (2.41)	
Anesthetist	Yes	41 (41)	4.49 (1.93)	0.34
	No	59 (59)	4.83 (2.17)	

*SD: standard deviation; ^†^: Wilcoxon-Mann-Whitney test.

Table 3 shows the activities professionals considered to be the nurses’ assignments during induction and reversal of anesthesia, with emphasis on patient identification and surgical positioning during induction. As for the reversal, nurses mentioned the Systematization of Perioperative Nursing Care (*SAEP*) and shift change.

**Table 3.** Main assignments cited by nurses during anesthesia induction and reversal. São Paulo-SP, Brazil, 2019.

Assignments in induction	n	%
Patient identification	85	85
Surgical positioning	79	79
Peripheral venous access puncture	75	75
Ventilation/supply of laryngoscope and tube	72	72
Recording of complications	71	71
Patient monitoring	70	70
Performance of Sellick/BURP* maneuvers	60	60
Patient education	60	60
Terms checking	60	60
**Reversal assignments**		
Perform SAEP^†^	85	85
Shift change	75	75
Patient transfer from the operating table	66	66
Aspiration assistance	62	62
Recording of drains, dressings	59	59

*BURP: back-up-right pressure; ^†^: *SAEP*: systematization of perioperative nursing care.

In the knowledge analysis, among the nurses evaluated, 77 (77%) correctly cited the periods of anesthesia, 84 (84%) the types of regional anesthesia, and 52 (52%) the types of regional anesthesia. Among the main predictors of difficult airway (DA), nurses highlighted short and wide neck (81; 81%) and limited head flexion/extension (79; 79%) (Table 4).

**Table 4.** Nurse’s knowledge about anesthesia (n = 100). São Paulo-SP, Brazil, 2019.

Variable		n	%
Periods of general anesthesia are induction, maintenance, and reversal	Yes	77	77
	No	13	13
Types of regional anesthesia are subarachnoid, epidural, and blocks	Yes	84	84
	No	16	16
Types of general anesthesia: total intravenous, inhalation, balanced	Yes	52	52
	No	48	48
**Difficult airway (DA) predictors**			
Short and wide neck		81	81
Head flexion/extension limitation		79	79
Mouth opening <3 cm		77	77
Mallampati classification >2		65	65

Among the participants, 97 (97%) reported that the hospital where they worked had materials available for difficult airways, especially straight laryngoscope blade (75%), laryngeal mask (73%), and bougie guide (72%).

Regarding patient safety, 87 (87%) nurses reported that the workplace had an adverse event reporting system, with 70 (80.45%) always reporting the occurrence of an adverse event and 56 (65.11%) reporting the incident even in the absence of harm to the patient. Table 5 shows the limiting factors for reporting the adverse events in anesthesia by nurses, according to the number of rooms under their responsibility.

**Table 5.** Limiting factors for reporting the adverse events in anesthesia and number of rooms under the nurse’s responsibility (N = 100). São Paulo-SP, Brazil, 2019.

Limitation for reporting the adverse event		N (%)	Number of rooms Mean (SD*)	p^†^
Work overload	Yes	43 (43)	4.98 (2.02)	0.18
	No	57 (57)	4.47 (2.21)	
Disbelief in the possibility of improvements	Yes	25 (25)	4.44 (2.22)	0.47
	No	75 (75)	4.77 (2.03)	
Communication difficulty	Yes	24 (24)	4.33 (2.26)	0.48
	No	76 (76)	4.80 (2.01)	
Absence of confidentiality	Yes	16 (16)	4.31 (1.85)	0.38
	No	84 (84)	4.76 (2.11)	
Lack of knowledge	Yes	12 (12)	4.42 (2.39)	0.65
	No	88 (88)	4.73 (2.04)	
Fear of punishment	Yes	9 (11)	3.36 (1.96)	0.02
	No	89 (89)	4.85 (2.04)	

*SD: standard deviation; ^†^: Wilcoxon-Mann-Whitney test.

The responses evaluated revealed ethical problems related to professional practice, with 47 (47%) nurses in the sample reporting that they sometimes remained in the operating room assisting the patient alone due to the absence of the anesthesiologist. Fifty-six (56%) reported that they had already administered anesthetics during the anesthetic procedure, 34 (34%) applied the anesthesia consent form, 10 (10%) performed intubation, and five (5%) performed extubation. Thirty-six (36%) nurses reported that in their services the anesthesiologist performed simultaneous care in more than two operating rooms, with an average of 1.53 rooms (SD = 0.88).

## DISCUSSION

The results indicated that several institutions in which nurses worked did not have a protocol to guide the team’s performance during anesthesia. Professionals recognized that nurses shall work during all periods of anesthesia; however, they face limitations for its practice due to the simultaneous performance of management and care activities, and insufficient staffing for the operating demand of the operating room, as well as gaps in knowledge regarding periods of general anesthesia and types of anesthesia. In addition, there were difficulties in reporting adverse events, the main reasons being the fear of being punished and reduced time for reporting events due to work overload.

The use of protocols to guide anesthesia work, such as checklists of care, indicated advances in the quality of care during the procedure in relation to the improvement of the work flow, effective communication between the nursing team and anesthesia team, decrease in adverse events, decrease in morbidity and mortality rates related to the anesthetic procedure^(12,13,14)^.

COFEN resolution No. 543/2017 recommends the dimensioning of one nurse for every three operating rooms, also considering the size of the surgeries performed in the institution^(15)^. However, in this study, the average nurse provided simultaneous daily care in more than four rooms, which was identified as a limiting factor for the performance of all activities assigned to them, especially assistance during periods of anesthesia, which was concentrated in the periods before and during anesthetic induction.

However, nurses recognized the implementation of SAEP as one of their main assignments in reversal, that is, only at the end of surgery and anesthesia, which seems to be the opposite, as *SAEP’s* principles reinforce its implementation in all stages of the perioperative period. In addition, this aspect is also noteworthy, since a large part of the sample reported responding only to the calls of the team in the operating room, that is, the professionals did not remain in the room during care provided to the surgical patient, which can hinder the continuity of care and care planning in the post-anesthetic recovery period.

Although *SAEP* is considered essential by perioperative nurses to ensure the quality of care, weaknesses are perceived regarding its implementation in clinical practice, possibly related to knowledge gaps regarding the steps of the nursing process, due to flaws in professional training and deficits in continuing education actions at work institutions^(16–17)^. In addition, the insufficient number of workers and the lack of adequate instruments for the complete and adequate application of *SAEP* can generate inconsistencies in its application process^(16–17)^.

Evidence shows that nurses defend a professional dimension that is more appropriate to care needs and that provides greater availability to provide direct care to surgical patients in the operating room, since a reduced number of nurses makes continuity of care planning and proper execution of SAEP difficult^(16,18–19)^. A higher proportion of nurses working in direct care was associated with better patient outcomes, better working conditions and hospital safety^(20)^.

The smaller number of rooms seemed to favor nurses’ participation in checking of anesthesia materials, an aspect that may be related to the fact that, with less commitment to the professional’s daily workload, there will be greater opportunity for direct care in the operating room, which includes planning anesthesia. Nursing service has a fundamental role in the care process, and the appropriate dimensioning of patients’ nursing care needs reflects an increase in nursing hours dedicated to care, contributing to health institutions achieving higher levels of quality of assistance and safe and humanized care^(21–22)^.

Knowledge gaps and doubts about the processes involving anesthesia can be limiting factors for nurses’ performance^(23)^, since the lack of clarity about the anesthesiologist’s care objectives impairs the nurses’ understanding of their role in ensuring the quality and safety of care in the anesthetic procedure. Team reflection on the context of their practice and the professionals’ working method create opportunities for improving work, communication, and achievement of common goals^(24)^.

The study showed that many professionals did not report non-damaging events, which does not allow for the correction of factors that can lead to the harmful event. Adverse events in anesthesia were related to the actions of professionals and the organizational structure, and involve failures in planning and executing tasks, weak communication, high workload, and pressure to perform tasks^(25)^.

*Near miss* may be associated, among other factors, with ineffective communication among professionals, failure to carry out an activity or lack of compliance with a protocol/guideline and a fragile institutional safety culture^(26)^. Institutional leaders shall promote a culture of organizational safety, in which failures can be identified and minimized before they cause harm to the patient, but which also promotes learning in terms of modifying care processes to avoid recurrences^(25)^.

Among the factors for not reporting adverse events, the fear of being punished was highlighted, which may be related to the professionals’ fear of exposing flaws in their care. Unfortunately, in disagreement with the movements to promote safety in health care, in many places a punitive culture still prevails, which predisposes to underreporting. Recognizing the value of notification reveals a potential for improving results, which can be achieved with investment in the team, through education, encouragement of notifications, and a management posture that reinforces a non-punitive culture^(27)^.

The dynamics of care for production, high workloads, and reduced staff in hospitals’ operating rooms can lead to care failures, but also to infractions in professional practice, such as those identified in this study, including the absence of an anesthesiologist or of its adequate proportion according to the number of rooms to be attended, or even the performance, by the nurses, of activities that are exclusive to the medical professional.

In accordance with the Federal Council of Medicine resolution No. 2.174/2017, simultaneous anesthesia in different patients, by the same professional at the same time, is prohibited^(28)^. Furthermore, the law of professional medical practice defines that tracheal intubation performance is exclusive to the physician^(6)^, and according to Resolution No. 2.174/2017 the application of the consent form for anesthesia is the anesthesiologist’s responsibility. Therefore, both physicians and nurses committed infractions in their daily activities and, consequently, exposed the patient’s safety to risks.

Thus, from a broader perspective, it is worth emphasizing the importance of the need for technical training of the nursing student in terms of contents related to perioperative care, as well as training for interdisciplinary work. In addition, strengthening of the actions of the class councils seems necessary, to supervise the professional practice and ensure that the nursing practice is in accordance with the standards and behaviors that are the responsibility of the profession. Finally, nursing specialty associations may and should contribute with the professionals’ continuing education actions for their continuous technical- scientific improvement and updating.

Limitations related to this study, such as the number of participants and their representation by region, can be pointed out, which restricted the analysis of working conditions in all Brazilian regions and may affect the generalization of the results. The option for electronic data collection may have influenced the professionals’ participation. These aspects raise the relevance of future research that include a greater number of professionals, allowing for a broad assessment of nurses’ working conditions in a Brazilian operating room.

## CONCLUSION

Nurses identified their role in the team during all periods of the anesthetic procedure, but they had difficulties in managing daily activities and continuity of care due to the execution of simultaneous activities and lack of personnel in the workplace. Among study participants, some professionals had deficient knowledge about periods and types of anesthesia.

Data revealed by this study seem to show some aspects to be evaluated, applied and adjusted in clinical practice, teaching and research.

As for clinical practice, the best professional dimensioning in hospitals is required to advance nursing care in anesthesia and to ensure its quality. Regarding teaching, nurses’ knowledge gaps regarding the anesthetic procedure can impair the quality of care, emphasizing the importance of improvements in professional qualification in perioperative nursing since their training and remaining during professional practice, through continuing education, to provide quality work, in accordance with current professional laws. Finally, broadening the discussion on the role of Brazilian nursing in anesthesia and the development of studies in anesthetic nursing may contribute to behavior uniformity and definition of its role, ensuring safer practices during the anesthetic procedure.
